# Addition to “Nanostars Carrying Multifunctional Neurotrophic Dendrimers Protect Neurons in Preclinical In Vitro Models of Neurodegenerative Disorders”

**DOI:** 10.1021/acsami.3c02300

**Published:** 2023-03-02

**Authors:** Corinne Morfill, Stanislava Pankratova, Pedro Machado, Nathalie K. Fernando, Anna Regoutz, Federica Talamona, Alessandra Pinna, Michal Klosowski, Robert J. Wilkinson, Roland A. Fleck, Fang Xie, Alexandra E. Porter, Darya Kiryushko

In the original version of this article (p. 47457), some acknowledgments were not included. In the revised Acknowledgments section provided below, we additionally provide The REC reference for the ethical approval of the human astrocyte isolation, an acknowledgment to Dr. Alize Proust at the Francis Crick Institute for establishing the triple coculture BBB model used in this study, and the reference and the grant number for the source of the human fetal material. This does not affect the results or conclusions of our work.

